# Obstruction of Photoinduced Electron Transfer from Excited Porphyrin to Graphene Oxide: A Fluorescence Turn-On Sensing Platform for Iron (III) Ions

**DOI:** 10.1371/journal.pone.0050367

**Published:** 2012-12-10

**Authors:** Zhong De Liu, Heng Xin Zhao, Cheng Zhi Huang

**Affiliations:** 1 Education Ministry Key Laboratory on Luminescence and Real-Time Analysis, Southwest University, Chongqing, People's Republic of China; 2 College of Pharmaceutical Sciences, Southwest University, Chongqing, People's Republic of China; 3 College of Chemistry and Chemical Engineering, Southwest University, Chongqing, People's Republic of China; Brandeis University, United States of America

## Abstract

A comparative reaserch of the assembly of different porphyrin molecules on graphene oxide (GO) and reduced graphene oxide (RGO) was carried out, respectively. Despite the cationic porphyrin molecules can be assembled onto the surfaces of graphene sheets, including GO and RGO, to form complexes through electrostatic and *π-π* stacking interactions, the more obvious fluorescence quenching and the larger red-shift of the Soret band of porphyrin molecule in RGO-bound states were observed than those in GO-bound states, due to the differenc of molecular flattening in degree. Further, more interesting finding was that the complexes formed between cationic porphyrin and GO, rather than RGO sheets, can facilitate the incorporation of iron (III) ions into the porphyrin moieties, due to the presence of the oxygen-contained groups at the basal plane of GO sheets served as auxiliary coordination units, which can high-efficiently obstruct the electron transfer from excited porphyrin to GO sheets and result in the occurrence of fluorescence restoration. Thus, a fluorescence sensing platform has been developed for iron (III) ions detection in this contribution by using the porphyrin/GO nanohybrids as an optical probe, and our present one exhibited rapid and sensitive responses and high selectivity toward iron (III) ions.

## Introduction

Graphene, a two-dimensional (2-D) nanomaterial consisting of sp^2^-hybridized carbon atoms forming a one-atom thick honeycomb lattice, exhibits remarkable electronic and mechanical properties [1]–[4]. Theoretically, the molecules of other allotropic carbon forms such as 1D carbon nanotubes (CNTs) and 0D fullerenes, can be built from graphene by rolling up or wrapping up graphene [5]. Thus, 2D graphene is widely used to describe the properties of various carbon-based materials. With the numerous reports of the many exceptional properties and applications of CNTs and fullerenes, the intensive research of graphene and its potential use as a nanometer-scale building block and fertile ground for analytical purpose, is expected. Especially, incorporation of light sbsorbing antenna chromophores through a covalent or noncovalent linkage with the extended π electrons of graphene sheets would constitute an ideal supramolecular nanoassembly, which has been considered to be important to tune optoelectronic properties of graphene [6]–[8] and enlarge the field of graphene-based analytical application [9]–[12].

As a kind of well-known functional dye, porphyrin derivatives, with a large extinction coefficient in the visible-light region, predictable rigid structure, and prospective photochemical electron-transfer ability, have been extensively used for modifying carbon nanomaterials (CNMs). A few studies have been reported for donor-acceptor-type nanohybrids, featuring the porphyrin derivatives as electron donor and the CNMs including fullene [13]–[15], carbon nanotubes [16]–[19] and reduced graphene oxide (RGO) [5], [20]–[23] as electron acceptor to generate charge-separated states. Quenching of the singlet excited molecules covalently grafted or supramolecularly assembled on the CNMs was observed in these nanohybrids through the electron transfer process.

**Figure 1:**
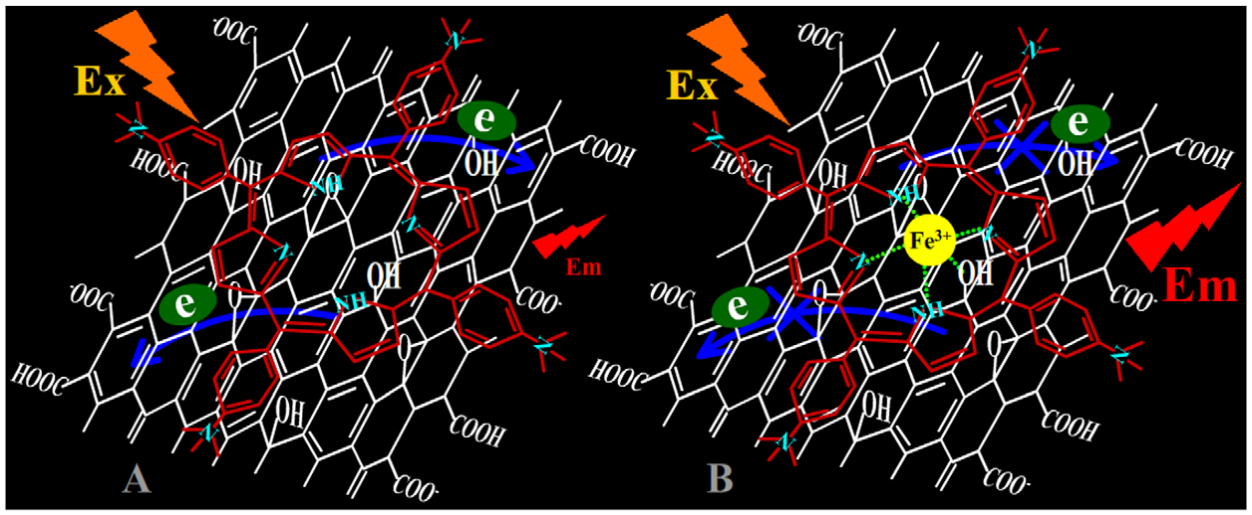
Schematic illustrations of PET from TAPP to GO sheets, and iron (III) ions selectively obstructing the process of PET.

Although many reports have investigated the interactive mechanism between porphyrin derivatives and graphene sheets, almost all these studies focus on the interactive affair between RGO and porphyrin derivatives [5], [20]–[23]. Therefore, in this contribution, a comparative reaserch of the assembly of different porphyrin molecules on graphene oxide (GO) and RGO was carried out, respectively. It was found that cationic porphyrin can be assembled onto the surfaces of graphene sheets, including RGO and GO, to form complexes through electrostatic and *π-π* stacking interactions. As a result, quenching of the singlet excited porphyrin molecules supramolecularly assembled on the graphene sheets was observed. However, besides existence of the difference of fluorescence quenching efficiency, the Soret band of porphyrin molecules in RGO-bound states shows a larger red-shift than those in GO-bound states, for the difference of molecular flattening on graphene sheets in degree. Further, more important finding was that not the RGO but the GO sheets, can facilitate the incorporation of iron (III) ions into the porphyrin moieties of porphyrin/graphene complexes, due to the presence of the oxygen-contained groups at the basal plane of GO sheets served as auxiliary coordination units. Consequently, the incorporation of iron (III) ions into the porphyrin moieties of porphyrin/GO complexes can obstruct the photoinduced electron transfer (PET) from excited porphyrin to GO sheets and result in the occurrence of fluorescence restoration. The PET and iron (III) ions selectively obstructing the process of PET from excited porphyrin to GO can be summarized in Figure 1A and 1B, respectively. The fluorescence turn-on sensing platform described here exhibited rapid and sensitive responses and high selectivity toward iron (III) ions.

## Materials and Methods

### Materials

Natural graphite powder (325 mesh) was purchased from Nanjing XFNANO Materials Tech Co., Ltd. (Nanjing, PRC). The GO and RGO was prepared and purified from natural graphite powder according to the modified Hummer's method [24], [25] and the procedures reported by Li and co-workers [26], respectively, and the obtained product was characterized by UV-visible spectra (Figure S1 in the Supporting Information). The α, β, γ, δ-tetrakis [4-(trimethylammoniumyl) phenyl] porphyrin (TAPP), 5, 10, 15, 20-tetrakis (1-methyl-4-pyridinio) porphyrin (TMPyP), and 5, 10, 15, 20-tetrakis (4-sulfopheny) porphyrin tetrasodium hydrate (TPPS_4_), ferritin and transferrin were purchased from Sigma-Aldrich. The human serum samples were collected from the individuals of healthy physical examination at the Hospital of Southwest University, in accordance to Institutional Review Board guidelines. All metal salts were of analytical grade and obtained from Beijing Chemical Reagent Co. Deionized water was used throughout the experiments, and all reagents described above were used as received without further purification.

### Apparatus

The fluorescence and the absorption spectra were recorded with a Hitachi F-2500 fluorescence spectrophotometer (Tokyo, Japan) and a Shimadzu UV-3600 spectrophotometer (Tokyo, Japan), respectively. The morphology of GO was observed on a Nanoscope Quadrex atom force microscope (Veeco, USA). X-ray photoelectron spectroscopy (XPS) analysis was obtained on an ESCRLRB250X (America) cispectrometer with a standard Al K source (1486.6 eV). The fluorescence lifetimes were measured with a FL-TCSPC fluorescence spectrophotometer (Horiba Jobin Yvon Inc, France). A Milestone Microwave Lab Stations was used to digest the real samples. Atomic absorption measurements were performed on TAS-990 atomic absorption spectrometer (AAS, Purkinje General Instrument Co., Ltd., Beijing). A Fangzhong pHS-3C digital pH meter (Chengdu, China) was used to measure the pH values of the aqueous solutions and a vortex mixer QL-901 (Haimen, China) was used to blend the solution.

### General Procedure

50 µl of porphyrin derivatives and 50 µl of HAc-NaAC (pH 4.1) buffer were at first pipetted into a 1.5-ml vial. Subsequently, 40 µl of 0.2 mg ml^−1^ GO was added and vortex-mixed. Then an appropriate volume of iron (III) ions working solution or sample solution was added, diluted to 500 µl with Milli-Q purified water and vortex-mixed thoroughly. The mixture was placed at least for 3 min and then transferred for absorbance and fluorescence measurements.

### Pretreatments of BioSamples

Real samples including two kinds of iron-contained proteins and serum were digested using a Milestone Microwave Lab Stations. In detail, an appropriate quantities of ferritin, transferrin and serum samples were combined with 7.0 ml of concentrated nitric acid and 1.0 ml of H_2_O_2_ (30%, w/w) in a Teflon digestion vessel. The vessels were placed in symmetrical positions on the turntable. The heating program was as follow: (1) ramp of 10 min up to 200°C; (2) maintenance 20 min at 200°C. After digestion, the samples were transferred into small beaker and heated to dry on a hot plate to remove the excessive acid and H_2_O_2_. Then the remaining residue was dissolved with purified water and used for the detection according to the general procedure and AAS measurements.

### Ethics Statement

The use of human subjects was approved by the University of Southwest's Institutional Review Board. A signed individual written informed consent agreement was obtained from the participants before beginning the work on this study, and the research didn't involve outside of our country of residence.

## Results and Discussion

### Supramolecular Assembly of Cationic Porphyrin on Graphene Sheets

**Figure 2:**
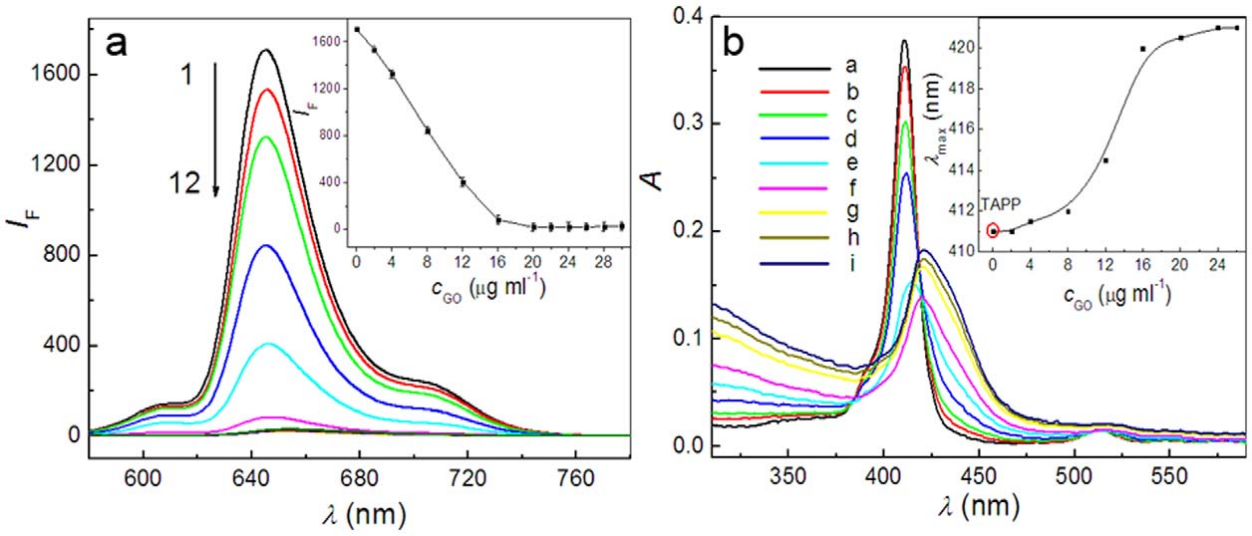
Fluorescence and absorption spectra recorded during addition of different concentrations of GO suspension to TAPP solution. The inset in Figure 2a shows that the variation of fluorescence intensity of TAPP at 645.0 nm varies with the increasing concentrations of GO. Concentrations: TAPP, 2.4 µM; GO from curve 2 to 12 (µg ml^−1^), 2.0, 4.0, 8.0, 12.0, 16.0, 20.0, 22.0, 24.0, 26.0, 28.0, 30.0. *λ*_ex_, 413.0 nm. The inset in Figure 2b shows that the variation of maximum absorption wavelength (*λ*_max_) of TAPP varies with the addition of GO. Concentration: TAPP, 2.4 µM; GO from curve b to i (µg ml^−1^), 2.0, 4.0, 8.0, 12.0, 16.0, 20.0, 24.0, 26.0. pH, 4.1.

**Figure 3:**
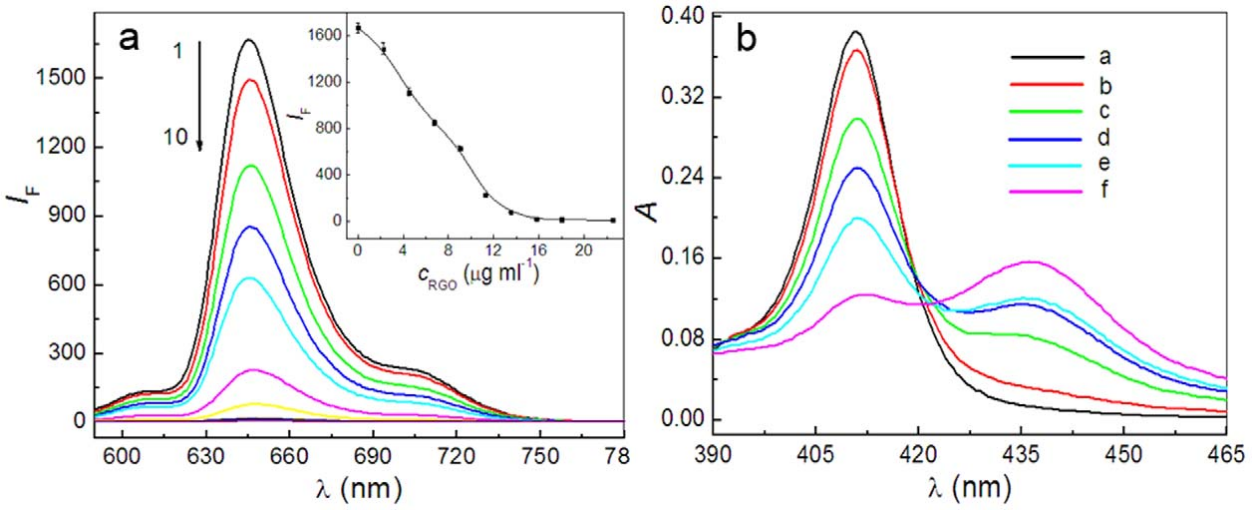
Fluorescence and absorption spectra recorded during addition of different concentrations of RGO suspension to TAPP solution. The inset in Figure 3a shows that the variation of fluorescence intensity of TAPP at 645.0 nm varies with the increasing concentrations of RGO. Concentrations: TAPP, 2.4 µM; RGO from curve 2 to 10 (µg ml^−1^), 2.2, 4.5, 6.8, 9.0, 11.2, 13.5, 15.8, 18.0, 22.5. *λ*_ex_, 413.0 nm. The concentration of RGO from curve b to f in Figure 3b (µg ml^−1^), 2.2, 4.5, 6.8, 9.0, 13.5. pH, 4.1.

Both RGO and GO sheets in aqueous dispersion are negatively charged because of their residual carboxyl groups and therefore can be viewed as a 2D anionic conjugated polymer. Thus, positively charged porphyrin molecules can be assembled onto the surfaces of graphene sheets to form complexes through electrostatic and π-π stacking interactions. As expected, the fluorescence of cationic porphyrin molecules, TAPP and TMPyP, were greatly quenched with the increasing concentrations of GO (Figure 2a, Figure S2a) and the quenching efficiency were calculated to be 95% and 76% for the same concentration of TAPP and TMPyP when the concentration of GO reaches 16 µg ml^−1^ (the inset in Figure 2a, Figure S2a). However, the fluorescence of anionic porphyrin molecules, TPPS_4_ was almost not quenched with the addition of different concentration of GO (Figure S3a). Furthermore, if RGO instead of GO was used in the process of assembly, an obvious fluorescence quenching of TAPP was also observed and a 99% quenching efficiency was obtained (Figure 3a).

The results described above were further supported by UV-visible spectra. As shown in Figure 2b, the spectrum of TAPP features an intense Soret band at 411 nm, and during the titration process of GO, the intensity of the original Soret band at 411 nm decreased gradually and a new Soret band at 427 nm appeared (the inset of Figure 2b). Similar phenomena were observed in the assembly process of TMPyP molecules on GO sheets and the maximum shift of the Soret band reached 15 nm (Figure S2b). However, there are no appreciable spectral changes in UV-visible spectra upon titration of TPPS_4_ solution with GO (Figure S3b), indicating that electrostatic attraction play essential role in the supramolecular assembly of porphyrin molecules on graphene sheets. Meanwhile, when RGO instead of GO was used in the process of assembly, the Soret band of the TAPP in RGO-bound states exhibits a larger red-shifts (27 nm) and a broader half-bandwidth than that of TAPP in GO-bound states (Figure 3b).

The difference of fluorescence quenching efficiency and absorption spectra make it clear that TAPP molecules have the stronger interactions with RGO than those with GO. Generally, compared with the GO, the RGO sheets share the feature of greater delocalized and conjugated electron structures due to chemical reduction [21], [22]. Therefore, the TAPP molecules have greater degree of molecular flattening on RGO than those on GO sheets, upon which the red-shift of the porphyrin Soret band depends according to the molecular flattening mechanism reported by Xu et al [20]. More concretely, in the structure of an unstrained TAPP molecule, four cationic trimethylammoniophenyl moieties are nearly perpendicular to the plane of porphyrin because of a strong steric hindrance [27]. When the trimethylammoniophenyl substituents rotate toward the coplanar conformation with respect to the porphyrin ring caused by molecular flattening on graphene sheets, the π conjugation and electron-withdrawing effect of TAPP will be enhanced [20]. As a result, more obvious fluorescence quenching and larger red-shift of the Soret band of TAPP in RGO-bound states were observed than those in GO-bound states, due to the differenc of molecular flattening in degree.

**Figure 4:**
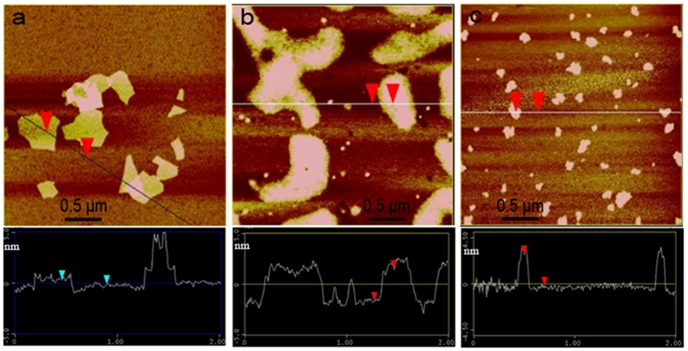
AFM images of GO, TAPP/GO complex and that in presence of iron (III) ions on mica substrate together with section analysis along the scored line. (a) GO; (b) TAPP/GO complex; (c) TAPP/GO complex in presence of iron (III) ions. Concentration: GO, 16.0 µg ml^−1^; TAPP, 2.4 µM; iron (III) ions, 8.0 µM.

On the basis of the spectral results described above, it is reasonable to conclude that the assembly of cationic porphyrin molecules on graphene sheets is triggered by electrostatic attraction, and facilitated by the π-π stacking cooperative interaction because the latter can further enlarge the π conjugation of porphyrin system and reduce the distance between the porphyrin plane and graphene sheets. Additionally, the AFM images also provided solid evidence for the molecular assembly of cationic porphyrin on GO sheets. As shown in Figure 4, the average thickness of a single-layer GO sheet was measured to be approximately 1.0 nm, which is consistent with the result reported previously [26], [28]. In comparison, the average thickness of a TAPP/GO complex sheet was determined to be about 1.6 nm with 0.6 nm increment compared with that of a pure GO sheet. Considering the thickness of one porphyrin molecule is about 0.5 nm [20], we can conclude that the TAPP molecules were adsorbed on GO sheets as a monolayer or submonolayer, not aggregates.

### The PET Process from Excited Porphyrin to GO Sheets as Selectively Obstructed by Iron (III) Ions

**Figure 5:**
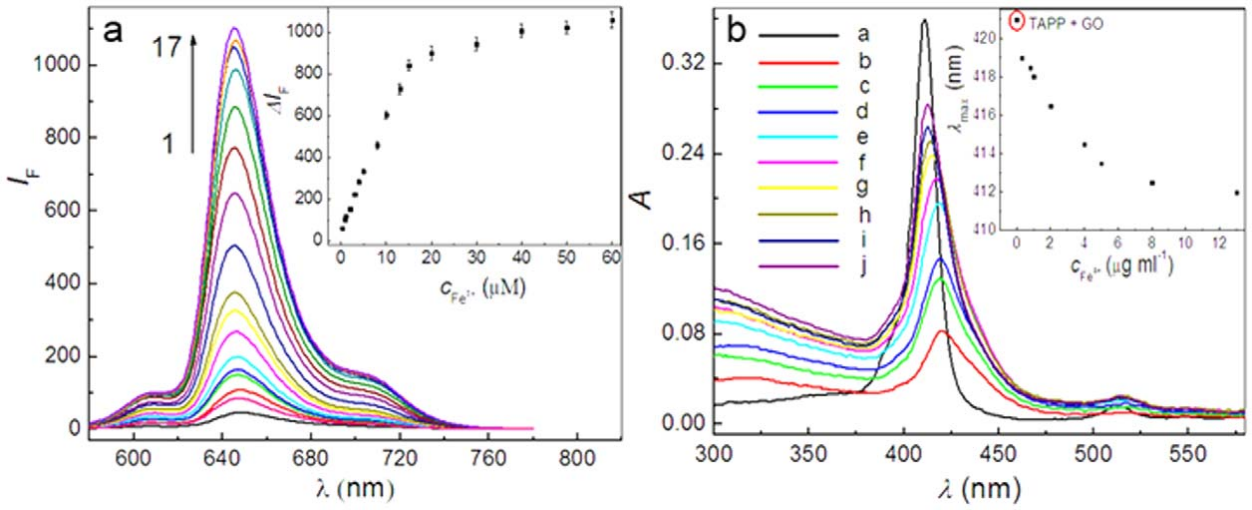
Fluorescence and absorption spectra recorded during addition of the increasing concentrations of iron (III) ions to TAPP/GO complex solution. The inset in Figure 5a shows that the enhanced fluorescence intensity at 645.0 nm varies with the increasing concentrations of the iron (III) ions. Concentration: TAPP, 2.4 µM; GO, 16.0 µg ml^−1^; iron (III) ions from curve 2 to 17 (µM), 0.3, 0.8, 1.0, 2.0, 3.0, 4.0, 5.0, 8.0, 10.0, 13.0, 15.0, 20.0, 30.0, 40.0, 50.0, 60.0. *λ*_ex_, 413.0 nm. The inset in Figure 5b is to intuitively display the blue-shift of Soret band of TAPP in GO-bound state with the addition of increasing concentration of iron (III) ions. Concentration: TAPP, 2.4 µM; GO except for curve a (µg ml^−1^), 16.0; iron (III) ions from curve c to j (µM), 0.3, 0.8, 1.0, 2.0, 4.0, 5.0, 8.0, 13.0. pH, 4.1.

More interestingly, the subsequent experiments confirmed that iron (III) ions can high-efficiently obstruct the process of PET from excited porphyrin to GO, but not from excited porphyrin to RGO sheets. As shown in Figure 5a and 5b, the fluorescence restoration and the blue-shift of Soret band of TAPP/GO complex occurred in presence of iron (III) ions, and more intuitionistic variations of the fluorescence enhancement (*ΔI*_F_) and the Soret band shift with the increasing concentration of iron (III) ions were displayed in the inset picture in Figure 5a and 5b, respectively. A maximum efficiency of fluorescence restoration can be calculated to reach 68%. Similar experimental phenomena also occurred to the TMPyP/GO complex when the iron (III) ions were introduced (Figure S4a and S4b in supporting information).

**Figure 6:**
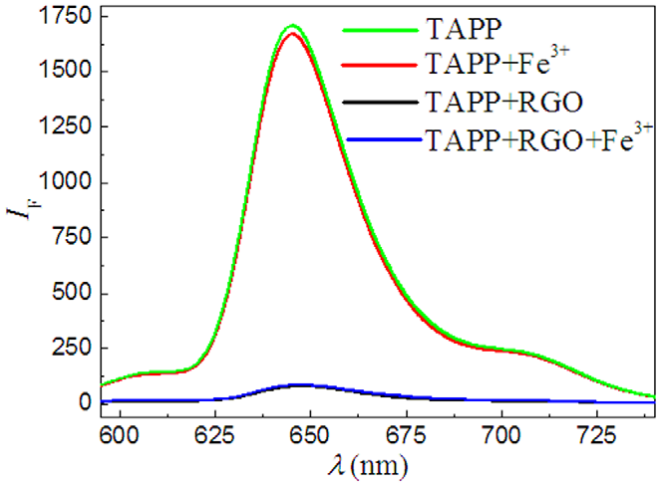
Fluorescence spectra of the TAPP, TAPP/RGO complex and that in presence of iron (III) ions. Concentrations: TAPP, 2.4 µM; RGO, 13.5 µg ml^−1^; iron (III) ions, 60.0 µM. *λ*_ex_, 413.0 nm, pH, 4.1.

Generally, the incorporation reaction of metal ions into the porphyrin ring is difficult due to the difficulty in deforming porphyrin ring [20], [29]–[30]. However, on the basis of forming TAPP/GO nanohybrids, the iron (III) ions being chelated by the TAPP moieties is very fast (Figure S5 in supporting information). We suppose that the oxygen-contained groups at the basal plane of GO sheets served as auxiliary coordination units might overcome this issue to some extent, and could facilitate the incorporation of iron (III) ions into the TAPP moieties, which can high-efficiently obstruct the process of PET from excited porphyrin to GO sheets and result in the occurrence of fluorescence restoration. In addition, we also noted that the fluorescence of pure TAPP molecules and TAPP/RGO complex was almost not an appreciable change in presence of iron (III) ions as shown in Figure 6, which also indirectly supported the mechanism due to the basal plane of RGO being lack of the oxygen functional groups. Further, the AFM image (Figure 4c) displayed that the average thickness of TAPP/GO complex sheet further increased to be about 2.2 nm in presence of iron (III) ions, which has also supported this contention as mentioned above.

**Figure 7:**
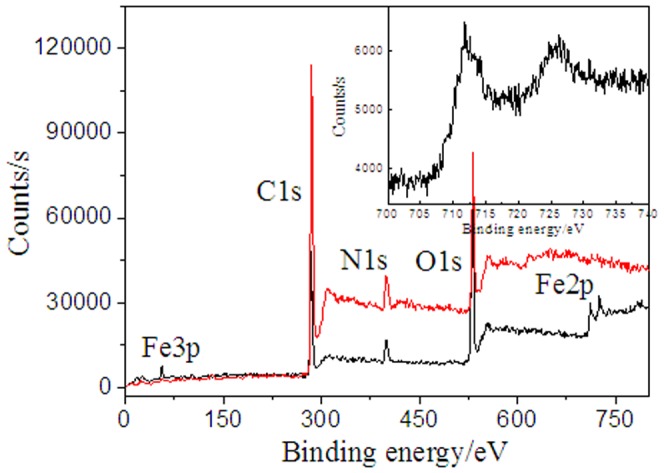
XPS wide-scan survey of the fresh obtained TAPP/GO (black line) and TAPP/RGO (red line) complexes exposed to the contained iron (III) ions solution, respectively. Inset shows detailed XPS survey of the Fe 2p region.

To intuitively confirm that not the RGO but the GO sheets, can facilitate the incorporation of iron (III) ions into the porphyrin moieties, we further performed the XPS analysis using fresh obtained TAPP/GO and TAPP/RGO complexes exposed to the contained iron (III) ions solution, respectively, through repeated centrifugation and washing steps. As shown in Figure 7, the XPS wide-scan of freshly prepared TAPP/GO complexes (black line) obviously reveal that in the presence of GO sheets, the iron (III) ions indeed was chelated by the TAPP moieties and formed three element complexes, TAPP/Fe^3+^/GO, compared with that of TAPP/RGO complexes (red line) dealed with the iron (III) ions. Besides a single photoelectron peak at ∼56 eV, corresponded to the binding energy of Fe 3p, the photoelectron peaks at ∼711 and ∼725 eV corresponded to the binding energies of 2p_3/2_ and 2p_1/2_ of iron (III) ions, respectively, were also observed [31]–[32], and more detailed XPS survey for Fe 2p regions were displayed in the inset picture in Figure 7, providing solid evidence for the formation of TAPP/Fe^3+^/GO complexes.

### Time-Resolved Emission Decay

**Figure 8:**
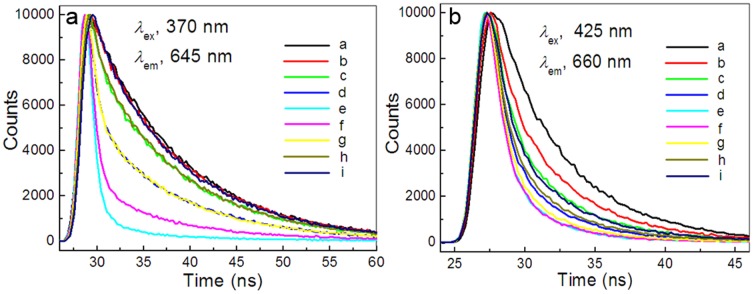
Time-resolved fluorescence Decays of the TAPP and TMPyP recorded at different GO concentrations and iron (III) ions. Concentration: TAPP, 2.4 µM, TMPyP, 3.0 µM; GO from curve b to i (µg ml^−1^), 8.6, 13.0, 17.3, 21.6, 21.6, 21.6, 21.6, 21.6, 21.6; iron (III) ions from curve f to i (µM), 2.0, 4.0, 8.0, 16.0. pH, 4.1.

The excited dynamics was elucidated by monitoring the emission decay time profiles of TAPP and TMPyP in the presence of different concentrations of GO and iron (III) ions. As shown in Figure 8, the fluorescence lifetime of porphyrin entities in the nanohybrids exhibited quick decays with the increasing concentrations of GO (Curve b, c, d, e) as compared with the ones without GO (Curve a). However, introducing the increasing concentrations of iron (III) ions into the TAPP/GO or TMPyP/GO solution, the fluorescence lifetime of porphyrin entities exhibited gradual restoration (Curve f, g, h, i). Notably, the shortening and prolonging of the fluorescence lifetimes nicely followed the steady-state fluorescence intensity quenching and restoring with the addition of GO and iron (III) ions in order, respectively.

A further observation is that the emission of singlet excited TAPP and TMPyP monitored in the absence of GO followed the single exponential decay with a lifetime of 9.57 and 5.34 ns, respectively. However, in the presence of GO and iron (III) ions, the decay trace deviates from the single exponential behavior and can be fitted with two exponential decay function (for fitting parameters see Table S1 in the Supporting Information). Kinetic parameters obtained during the fitting procedure revealed that long and short time components, attributed to the excited state of TAPP and TMPyP in the unbound and the GO-bound states, respectively, appeared in the system. More interesting is that the ratios of pre-exponential factor B_1_/B_2_, represented the contribution of the components with short lifetime to the overall decay, increased with increasing GO concentration and rapidly decreased with increasing iron (III) ions concentration. The results also have further supported that the GO sheets can quenched the fluorescence of cationic porphyrin by the electron transfer mechanism and the iron (III) ions can obstruct the process of PET by incorporation into the moieties of porphyrin in GO-bound states.

### Sensitive and Selective Detection of Iron (III) Ions

Under the optimal pH conditions (Figure S6 in Supporting Information), different concentrations of iron (III) ions were used to construct the calibration curves. The enhanced fluorescence intensities (*ΔI*_F_) at 645.0 nm can be fitted as the equation of *ΔI* = 60.7+52.2 *c* (iron (III) ions, 10^−6^ M) over the range of 3.0×10^−7^ to 1.5×10^−5^ M with the correlation coefficient of 0.9988 and the limit of determination (3*σ*) of 10 nM.

**Figure 9:**
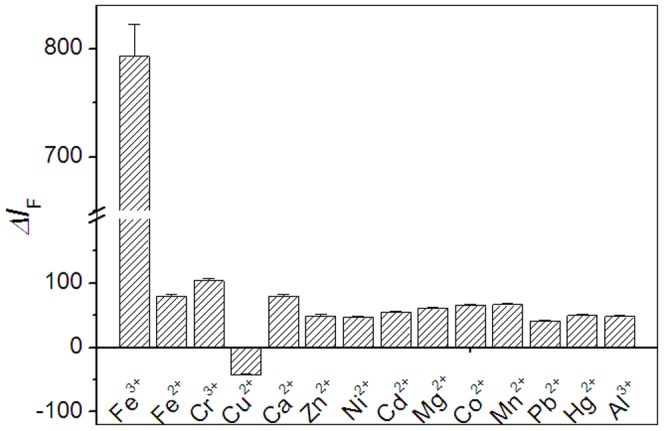
Fluorescence response of TAPP/GO complex to various metal ions. Concentration: TAPP, 2.4 µM; GO, 16.0 µg ml^−1^; iron (III) ions, 15.0 µM; Other metal ions were all 30.0 µM. All data were collected at 645.0 nm.

The selectivity of the TAPP/GO complexes as an optical probe toward iron (III) ions was also studied. The enhanced fluorescence intensities (*ΔI*_F_) were plotted against various metal ions, including Fe^2+^, Cr^3+^, Cu^2+^, Ca^2+^, Zn^2+^, Ni^2+^, Cd^2+^, Mg^2+^, Co^2+^, Mn^2+^, Pb^2+^, Hg^2+^ and Al^3+^. As shown in Figure 9, although the used concentration of other metal ions is two times than the one of iron (III) ions, the optical response of the probe toward iron (III) ions is about 10 times than those toward them. It is worth mentioning that the optical response of TAPP/GO complexes toward Cu^2+^ ions displayed a negative value, which was attributed to the Cu^2+^ ions itself quenching the fluorescence of TAPP molecules. In other words, the Cu^2+^ ions, to some extent, would exert the influence on the measurement of iron (III) ions. However, compared with the iron element as essential micronutrients for normal organism, the content of Cu^2+^ ions, one of all kinds of toxic heavy metal ions, is very low in real biological samples and not sufficient to cause any interference.

In order further to identify this point, we detected the concentration of iron (III) ions in both serum samples and two kinds of iron-contained proteins using our present method since the species are most common and important ions in biological systems. The results and recoveries resulting from the average of three determinations were summarized in Table 1. As shown in Table 1, such iron (III) ions assay displays a high selectivity towards iron (III) ions against a background of complicate biological samples, and the results show good agreement with the found values determined by atomic absorption spectrometry. Our present contribution has showed that the donor-acceptor-type nanohybrides could find their analytical applications in complicate samples based on a fluorescence turn-on strategy, which could supply a new train of thought and fertile ground for analytical purpose.

**Table 1:** Determination of iron (III) ions in the iron-contained protein and human serum sample using the proposed method and AAS.

Sample	Iron (III) ions (µM)				
	Proposed method Mean^a^+SD^b^	AAS Mean^a^+SD^b^	Added	Found Mean^a^+SD^b^	Recovery (%)
Ferritin	8.5±0.4	8.4±0.3	5.0	13.4±0.4	90.6∼106.2
Transferrin	5.2±0.3	5.3±0.2	5.0	10.3±0.4	94.4∼110.3
Serum	9.7±0.4	9.8±0.3	5.0	14.8±0.5	92.8∼112.0

a Mean of three determinations.

b SD, standard deviation. Concentration: TAPP, 2.4 µM; GO, 16.0 µg ml^−1^; pH 4.1.

## Conclusion

In conclusion, two points should be emphasized. First, cationic porphyrin molecules can be assembled onto the surfaces of graphene sheets, including GO and RGO, to form complexes through electrostatic and π-π stacking interactions. During the process of assembly, it is the electrostatic attraction that play essential role, and the π-π stacking cooperative interaction could further play a promoting role. Consequently, the cationic porphyrin molecules have the stronger interactions with RGO than those with GO. Second, on the basis of forming complexes between cationic porphyrin and GO, but not RGO sheets, the iron (III) ions can further incorporate into the porphyrin ring to form three element composite materials and obstruct the process of PET from porphyrin to GO, in which the oxygen-contained groups at the basal plane of GO sheets served as auxiliary coordination units is indispensable and main driving forces for the incorporation of iron (III) ions.

Based on the above findings, a fluorescence turn-on strategy has been developed for detection of iron (III) ions in complicate samples using the TAPP/GO nanohybrids as an optical probe. The approach described here exhibited rapid and sensitive responses and high selectivity toward iron (III) ions. Besides, the properties that the iron (III) ions can high-efficiently obstruct the process of PET from excited porphyrin to GO could be potential value in the areas of designing novel energy conversion architectures.

## Supporting Information

Figure S1 Absorption spectra of GO and RGO dispersed in water. The maximum peak shifts from 228 nm to 266 nm after reduction, implying the electronic conjugation within the graphene sheets is restored due to hydrazine reduction.
(TIF)

Figure S2 Fluorescence and absorption spectra recorded during addition of different concentrations of GO suspension to TMPyP solution. The insets in Figure S2a and S2b show that the variation of fluorescence intensity at 660.0 nm and maximum absorption wavelength (*λ*_max_) of TMPyP varies with the increasing concentrations of GO, respectively. Concentrations: TMPyP, 2.4 µM; GO (µg ml^−1^) from curve 2 to 9 (curve b to i), 2.0, 4.0, 8.0, 12.0, 16.0, 20.0, 24.0, 28.0. *λ*_ex_, 425.0 nm, pH, 4.1.
(TIF)

Figure S3 Fluorescence and absorption spectra of the TPPS_4_ in absence and presence of different concentrations of GO. Concentrations: TPPS_4_, 2.4 µM; GO from curve b to g (µg ml^−1^), 4.0, 8.0, 12.0, 16.0, 20.0, 24.0. *λ*_ex_, 430.0 nm, pH, 4.1.
(TIF)

Figure S4 Fluorescence and absorption spectra recorded during addition of the increasing concentrations of iron (III) ions to TMPyP/GO complex solution. The inset in Figure S4a shows that the enhanced fluorescence intensity at 660.0 nm varies with the increasing concentrations of the iron (III) ions. *λ*_ex_, 425.0 nm. The inset in Figure S4b is to intuitively display the blue-shift of Soret band of TMPyP in GO-bound state with the addition of increasing concentration of iron (III) ions. Concentration: TMPyP, 2.4 µM; GO except for curve a, 16.0 µg ml^−1^; iron (III) ions (µM) from curve 2 to 8 (curve c to i), 1.0, 3.0, 6.0, 8.0, 12.0, 16.0, 20.0. pH, 4.1.
(TIF)

Figure S5 Effect of time on the fluorescence intensity of TAPP/GO nanohybrids recorded in the absence (black line) and presence (red line) of iron (III) ions. Concentration: TAPP, 2.4 µM; GO, 16.0 µg ml^−1^; iron (III) ions, 10.0 µM.
(TIF)

Figure S6 Dependence of the fluorescence intensity on the pH of the system. Concentration: TAPP, 2.4 µM; GO, 16.0 µg ml^−1^; iron (III) ions, 15.0 µM.
(TIF)

Table S1 Parameters describing decay of the TAPP and TMPyP fluorescence during addition of increasing concentrations of GO and iron (III) ions, respectively.
(TIF)

Text S1 Detailed Procedure for Synthesis and Purification of Graphene Oxide and Reduced Graphene Oxide.
(DOC)
