# Supplementary figures and images for: Obstruction of Photoinduced Electron Transfer from Excited Porphyrin to Graphene Oxide: A Fluorescence Turn-On Sensing Platform for Iron (III) Ions
Source: PLoS One. 2012 Dec 10;7(12):e50367. doi: 10.1371/journal.pone.0050367 (PMC3519470; doi:10.1371/journal.pone.0050367)

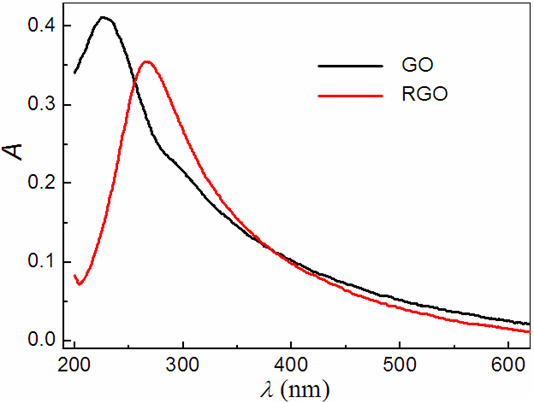

Supplement: Figure S1 — Absorption spectra of GO and RGO dispersed in water. The maximum peak shifts from 228 nm to 266 nm after reduction, implying the electronic conjugation within the graphene sheets is restored due to hydrazine reduction. (TIF) [file pone.0050367.s001.tif]

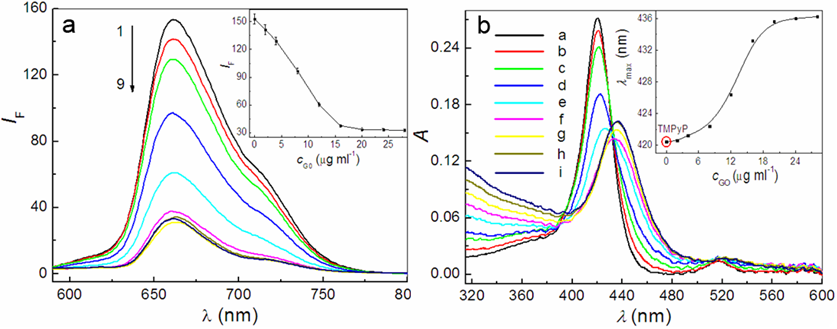

Supplement: Figure S2 — Fluorescence and absorption spectra recorded during addition of different concentrations of GO suspension to TMPyP solution. The insets in Figure S2a and S2b show that the variation of fluorescence intensity at 660.0 nm and maximum absorption wavelength (λ max) of TMPyP varies with the increasing concentrations of GO, respectively. Concentrations: TMPyP, 2.4 µM; GO (µg ml−1) from curve 2 to 9 (curve b to i), 2.0, 4.0, 8.0, 12.0, 16.0, 20.0, 24.0, 28.0. λ ex, 425.0 nm, pH, 4.1. (TIF) [file pone.0050367.s002.tif]

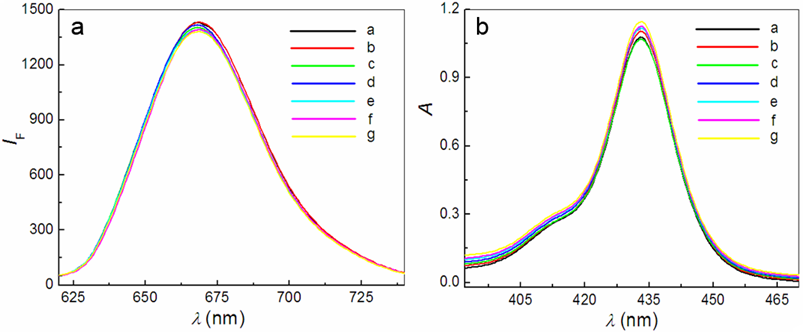

Supplement: Figure S3 — Fluorescence and absorption spectra of the TPPS4 in absence and presence of different concentrations of GO. Concentrations: TPPS4, 2.4 µM; GO from curve b to g (µg ml−1), 4.0, 8.0, 12.0, 16.0, 20.0, 24.0. λ ex, 430.0 nm, pH, 4.1. (TIF) [file pone.0050367.s003.tif]

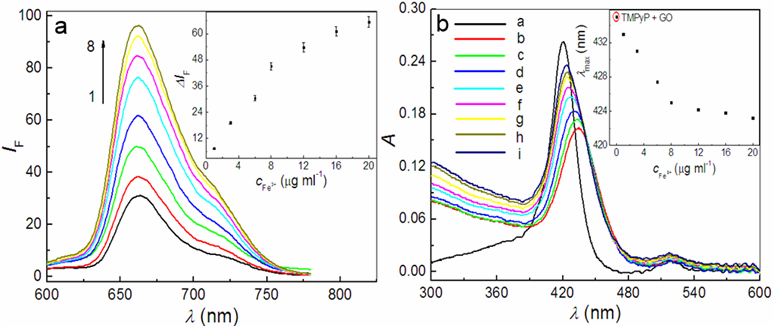

Supplement: Figure S4 — Fluorescence and absorption spectra recorded during addition of the increasing concentrations of iron (III) ions to TMPyP/GO complex solution. The inset in Figure S4a shows that the enhanced fluorescence intensity at 660.0 nm varies with the increasing concentrations of the iron (III) ions. λ ex, 425.0 nm. The inset in Figure S4b is to intuitively display the blue-shift of Soret band of TMPyP in GO-bound state with the addition of increasing concentration of iron (III) ions. Concentration: TMPyP, 2.4 µM; GO except for curve a, 16.0 µg ml−1; iron (III) ions (µM) from curve 2 to 8 (curve c to i), 1.0, 3.0, 6.0, 8.0, 12.0, 16.0, 20.0. pH, 4.1. (TIF) [file pone.0050367.s004.tif]

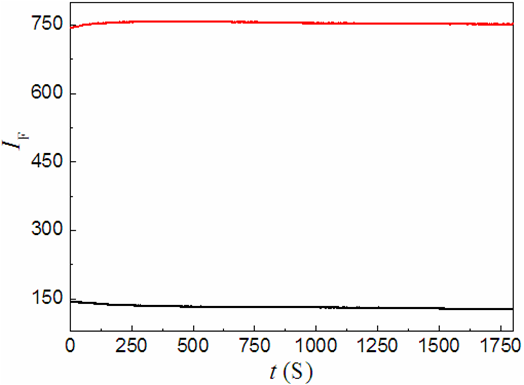

Supplement: Figure S5 — Effect of time on the fluorescence intensity of TAPP/GO nanohybrids recorded in the absence (black line) and presence (red line) of iron (III) ions. Concentration: TAPP, 2.4 µM; GO, 16.0 µg ml−1; iron (III) ions, 10.0 µM. (TIF) [file pone.0050367.s005.tif]

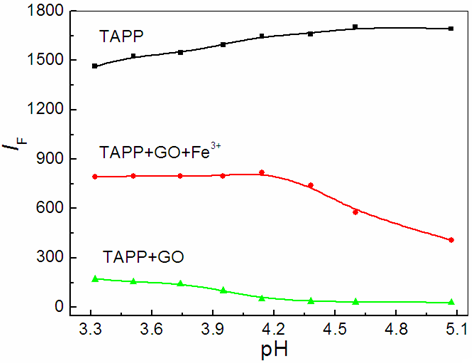

Supplement: Figure S6 — Dependence of the fluorescence intensity on the pH of the system. Concentration: TAPP, 2.4 µM; GO, 16.0 µg ml−1; iron (III) ions, 15.0 µM. (TIF) [file pone.0050367.s006.tif]

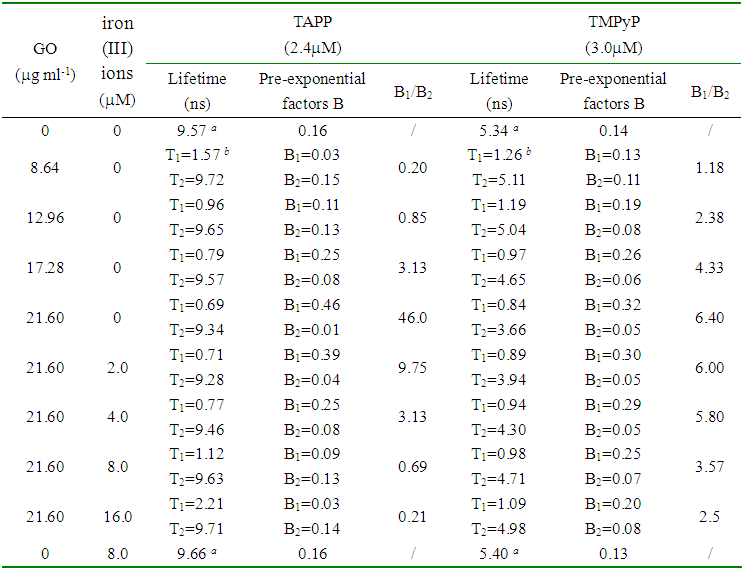

Supplement: Table S1 — Parameters describing decay of the TAPP and TMPyP fluorescence during addition of increasing concentrations of GO and iron (III) ions, respectively. (TIF) [file pone.0050367.s007.tif]
