# Supplementary material for: Obstruction of Photoinduced Electron Transfer from Excited Porphyrin to Graphene Oxide: A Fluorescence Turn-On Sensing Platform for Iron (III) Ions
Source: PLoS One. 2012 Dec 10;7(12):e50367. doi: 10.1371/journal.pone.0050367 (PMC3519470; doi:10.1371/journal.pone.0050367)
Supplement: Text S1 — Detailed Procedure for Synthesis and Purification of Graphene Oxide and Reduced Graphene Oxide. (DOC) [file pone.0050367.s008.doc]

**Supporting Information**

***for***

Obstruction of Photoinduced Electron Transfer from Excited Porphyrin to Graphene Oxide: A Fluorescence Turn-on Sensing Platform for Iron (III) Ions

Zhong De Liu^1, 2^ Heng Xin Zhao^3^ and Cheng Zhi Huang^*, 1, 2, 3^

1Education Ministry Key Laboratory on Luminescence and Real-Time Analysis, Southwest University, Chongqing, China, 2College of Pharmaceutical Sciences, Southwest University, Chongqing, China, 3College of Chemistry and Chemical Engineering, Southwest University, Chongqing, China

**Detailed Procedure for Synthesis and Purification of Graphene Oxide and Reduced Graphene Oxide.**

The GO and RGO was prepared and purified from natural graphite powder according to the previously reported literature [1-3], respectively, In detail, graphite powder (3.0g) was put into an 80 °C solution of concentrated H_2_SO_4_ (12.0 ml) containing P_2_O_5_ (2.5g), and K_2_S_2_O_8_ (2.5g) for 4.5 h. The mixture was cooled to room temperature, diluted with 500 ml of Milli-Q purified water and left overnight. Then, the mixture was filtered and washed with water using a 0.45 μm microporous membrane to remove the residual acid. The product was dried under room temperature overnight. Subsequently, these pre-oxidized graphite powder were put into cold (0 ^o^C) concentrated H_2_SO_4_ (120.0 ml). Then, KMnO_4_ (15.0g) was slowly added under stirring, and the temperature of the mixture was kept to be below 20 0 ^o^C in an ice-bath. Successively, the mixture was stirred at 35 ^o^C for 2 h and then diluted with water (250 ml) in an ice-bath to keep the temperature below 50 ^o^C. The mixture was stirred for 2 h, and then an additional 700 ml of water and 20 ml of 30% H_2_O_2_ were added. The mixture was filtered and washed with 1:10 HCl solution (1000 ml) to remove metal ions, and followed by 1000 ml of water to remove the acid. The resulting solid was dried at 50 ^o^C. Finally, it was purified by dialysis for one week to remove the remaining metal species.

As for the preparation of RGO, in briefly, fifty milliliters of purified GO dispersion (0.2 mg ml^-1^) was mixed with 15 μl of hydrazine monohydrate and 150 μl of ammonia solution (28% in water). The mixture was stirred at 95 °C for 1.5 h. After reduction, a homogeneous black dispersion with a small amount of black precipitate was obtained. Successively, this dispersion was filtered through glass cotton to remove the precipitate and yield a stable black aqueous dispersion of RGO, which was used to the comparative reaserch in subsequent experiments.

References

1 Hummers WS, Offeman RE (1958) Preparation of graphitic oxide. J. Am. Chem. Soc. 80: 1339.

2 Xu YX, Bai H, Lu GW, Li C, Shi GQ (2008) Flexible graphene films via the filtration of water-soluble noncovalent functionalized graphene sheets. J. Am. Chem. Soc. 130: 5856-5857.

3 Li D, Muller MB, Gilje S, Kaner RB, Wallace GG (2008) Processable aqueous dispersions of graphene nanosheets. *Nat. Nanotechnol.* *3*: 101-105.
